# A Quantum Blind Multi-Signature Method for the Industrial Blockchain

**DOI:** 10.3390/e23111520

**Published:** 2021-11-15

**Authors:** Zhengying Cai, Shi Liu, Zhangyi Han, Rui Wang, Yuehua Huang

**Affiliations:** 1 College of Computer and Information Technology, China Three Gorges University, Yichang 443002, China; lswy0221@163.com (S.L.); hzy571927306@163.com (Z.H.); wangrui_caff@hust.edu.cn (R.W.); 2 College of Electrical Engineering & New Energy, China Three Gorges University, Yichang 443002, China; hyh@ctgu.edu.cn

**Keywords:** industrial blockchain, anti-quantum, quantum blind multi-signature, multi-party transaction

## Abstract

Traditional anti-quantum methods and multi-signature technologies to secure the blockchain against quantum attacks will quickly reduce the efficiency and scalability of the industrial blockchain, where the computational resources will experience a polynomial rise with the increasing number of traders. Here, a quantum blind multi-signature method is proposed for the multi-party transaction to provide anti-quantum security. First, the proposed multi-party transaction frame and quantum key distribution in the industrial blockchain are introduced. It integrates a novel quantum blind multi-signature algorithm that is based on the quantum entanglement mechanism, and it is absolutely secure in theory. Second, the anti-quantum multi-signature algorithm is illustrated, where there are four phases, i.e., initialization, signing, verification, and implementation. Third, the security and complexity of the proposed framework are analyzed and compared with related methods in references, and our proposed method is verified to be able to offer good computational performance and blockchain scalability for multi-party transaction. Last, the paper is summarized and future research directions are proposed.

## 1. Introduction

Today, more and more researchers have noticed the blockchain technology and its industrial application. Industrial blockchain can bring secure architecture for modern industries, such as secure data storage and recovery [1]. With the help of blockchain, traditional industrial businesses and trades will be more secure [2]. However, most researchers have insisted that, as an emerging technology, quantum computers may threaten traditional cryptography technologies in blockchain through quantum mechanics [3]. In 2018, a paper in Nature speculated that quantum computers will put blockchain at risk [3], and then another work in Nature [4] claimed a programmable superconducting processor could get quantum supremacy.

Therefore, it is very necessary to improve the anti-quantum security of industrial blockchain [5]. The anti-quantum security of blockchain, also called post-quantum, quantum proof, quantum safe, or quantum resistance, means the coming of a post-quantum era, which is a new trend rising fast in the area of security and privacy protection of blockchain.

Recent anti-quantum technologies can be divided into two main categories. The one kind is the classic anti-quantum technologies, including the post-quantum smart contract [6], post-quantum cryptocurrency scheme [7], post-quantum multi-secret sharing [8], anti-quantum proxy blind signature [9], lattice-based signature scheme [10,11,12], etc. Although these classic anti-quantum technologies are promising to secure the blockchain architecture and digital assets, they suffer from algorithm complexity and environmental vulnerability along with the increasing scale of blockchain. For example, lattice-based cryptography needs to solve all kinds of complicated calculation problems on lattice, which are often NP-hard problems [10,11,12]. Hence, these classic anti-quantum technologies are considered be relatively safe.

The other kind of anti-quantum technology is the quantum technology itself. That is, it aims to improve the quantum resistance of blockchain by using quantum mechanics, including the quantum signature [13], quantum Fourier transfer [14], and quantum blind signature scheme [15], etc. This kind of anti-quantum technology utilizes the special mechanism of quantum mechanics and has nothing to do with algorithm complexity, so we consider them as an absolute safe.

Nevertheless, research studies on anti-quantum technologies of blockchain have neglected the multi-signature transactions, which are very popular in industrial blockchain [16,17]. Both classic anti-quantum technologies [6,7,8,9,10,11,12] and quantum anti-quantum technologies [13,14,15] cannot be directly used in the multi-signature transactions of blockchain to provide quantum resistance and blockchain scalability. Moreover, the existing multi-signature schemes in blockchain [16,17] cannot both provide anti-quantum security and keep the computational performance and the scalability of blockchain.

To deal with these issues, this paper proposes an anti-quantum blockchain framework based on quantum blind multi-signature method without an arbitrator to secure the blockchain against quantum attacks. The main contributions of this paper are as follows.

(1) A quantum blockchain framework is proposed to improve the quantum resistance of blockchain. Multiple traders can implement quantum signing and verification to complete a multi-party transaction. To the best of our knowledge, this is the first time to apply quantum blind multi-signature for industrial blockchain.
(2) A quantum blind multi-signature algorithm is designed to include four phases, i.e., initialization, signing, verification, and implementation. Furthermore, a blind message is employed in multi-party business to protect private information.
(3) The security and computational performance against quantum attacks of the proposed method are analyzed and compared. The proposed scheme can realize absolute security and good scalability and can be directly used in lightweight and decentralized multi-party transactions of blockchain.

The rest of this article is structured as follows. Section 2 reviews related work on anti-quantum technologies on blockchain. Section 3 introduces our anti-quantum blockchain and quantum key distribution for multi-party transactions. Section 4 explains the main phases for the proposed quantum blind multi-signature scheme and analyzes the algorithm flow and its computational performance. Section 5 compares the performance of the proposed method with other post-quantum signature algorithms. Finally, Section 6 summarizes the whole paper and indicates future directions.

## 2. Relevant Work

The industrial blockchain is the innovative application of blockchain technology to the industrial Internet and promotes the interconnection of everything and everything on the chain [1,2]. The actual business scenarios between enterprises often have a large number of trading objects. In large-scale and multi-participant business scenarios, the industrial blockchain can play a unique transformation potential of 1 + 1 > 2 for the digital upgrading of the industrial chain [16,17].

Before the application of blockchain, enterprises mostly used point-to-point trading methods, which have low transaction execution efficiency, and it is difficult to trace and track the transaction. Furthermore, the centralized database storage also makes its transaction data easy to be tampered with. However, the blockchain technology can greatly improve the trust between various industrial participants and avoid the trust risks by breaking the data island [18]. The industrial blockchain can further improve and optimize business processes, improve collaboration efficiency, reduce operation costs, and realize the construction of trusted system [19].

On the other hand, the blockchain technology has its own vulnerability to some attacks [20], such as the coin hopping attack [21], forgery attack [16], hyperledger access control [22], and especially quantum attack [3,4]. Until now, the popular cryptographic protocols used to secure the blockchain have proved to be subject to quantum attacks. The authors of [23] estimated that the proof-of-work in Bitcoin could be relatively resistant to the quantum computers in the next decade, but the elliptic curve signature in Bitcoin could be completely broken by a quantum computer in about 2027. The authors of [24] gave a slow defense for Bitcoin against a fast quantum computing attack.

Hence, the available post-quantum signature schemes are necessary to better meet the security requirements of future blockchain, where most are classic post-quantum signature methods. Because this kind of post-quantum signature schemes do not need to greatly change the classic architecture of the existing industrial blockchain, they have gained a lot of views, such as the post-quantum smart contract [6], post-quantum cryptocurrency scheme [7], post-quantum multi-secret sharing [8], anti-quantum proxy blind signature [9], lattice-based signature scheme [10,11,12], etc.

Lattice-based cryptography utilizes number-theoretical hard problems and has become the most popular method among all anti-quantum technologies, directly resulting in the lattice-based signature schemes [10,11,12]. To further resist the quantum attacks on the cryptographic algorithms in blockchain systems, the authors of [9] put forward an anti-quantum proxy blind signature algorithm based on the lattice cryptography to provide anonymity and untraceability, and the scheme was proved to be able to provide security in a random oracle model. The authors of [10] designed a lattice-based signature scheme to build a threshold-based post-quantum multi-secret sharing for a secure verifiable blockchain. Based on the lattice signature scheme, the authors of [25] stated a quantum-resistance authentication, the authors of [26] designed an identity-based anti-quantum privacy-preserving blind authentication, and the authors of [27] described a compiler for post-quantum primitives.

These classic post-quantum methods have proven to be effective to some extent. The authors of [28] analyzed the polymer-encapsulated molecular-doped epigraphene for quantum-resistance metrology, and the authors of [29] reviewed the hash-based signatures in the quantum-safe Internet of Things. With the help of these classic post-quantum technologies, the anti-quantum performance of blockchain can be improved at the expense of system performance, since these kinds of classic post-quantum methods need complex algorithms to improve the security. If a lot of classic post-quantum algorithms are run on industrial blockchain, the system performance and blockchain scalability will be greatly decreased.

The other kind of anti-quantum technologies has gained attention recently, namely quantum anti-quantum methods, such as the quantum signature [13], quantum Fourier transfer [14], and quantum blind signature scheme [15]. With the emergence of quantum computing, the quantum security and vulnerabilities of blockchain again attracted more and more analysis. The authors of [3] insisted that the Grover’s and Shor’s algorithms can destroy DES and RSA cryptography systems, which are popular in industrial blockchain.

Multi-signature and multi-party transactions are also important in the decentralized industrial blockchain [30]. To improve the signature security, the authors of [16] introduced a multi-signature technology into decentralized energy trading blockchains, and the authors of [17] described a multi-signature scheme for enterprise blockchain platforms. To protect the transaction privacy in multi-party business blockchain, the authors of [31] put forward an offline arbitrated quantum blind dual-signature protocol to resist existential forgery attacks, and it could provide absolute security of quantum signature and the anonymity of the signers. The authority of the arbitrator is reduced to be offline in [31], so that it is not compulsory for an arbitrator to verify the signatures. To resist quantum attacks, the authors of [8] considered a threshold-based multi-secret sharing for post-quantum secure blockchain.

However, the most quantum blind multi-signature method is not suitable for decentralized blockchain transactions. The blockchain-based secure solutions provide a distributed, verifiable ledger and a series of blocks, which are linked to finish a transaction and publish it in all the subsequent blocks. This kind of special architecture causes blockchain to be a double-sided sword. On the one hand, blockchain technology can improve the security of many industrial applications [1,2]. On the other hand, a complex cryptography system or multi-signature scheme will impair the performance and scalability of industrial blockchains. For example, the authors of [32] described a quantum-resistance authentication in centralized cognitive radio networks, which is not suitable for uncentralized blockchain architecture. Additionally, the quantum single signature [13], quantum Fourier transfer [14], and quantum blind signature scheme [15] are also not suitable to be directly applied into multi-signature businesses of blockchains.

## 3. Quantum Multi-Party Blockchain

### 3.1. Multi-Party Blockchain Transaction

**Figure 1:**
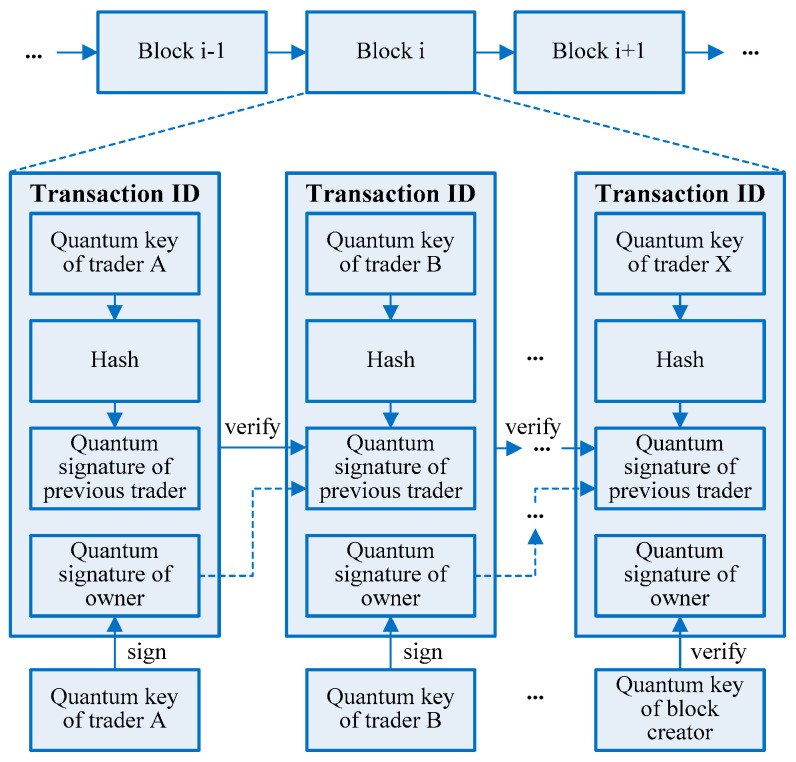
The proposed multi-party transaction mechanism in the industrial blockchain.

In a multi-party transaction of industrial blockchain, there are multiple traders [1,2,8], as shown in Figure 1. Every transaction is denoted as a unique transaction ID, where each trader should verify the previous quantum signature, and then make his quantum signature before sending the transaction message to the next trader [16,17,30,31]. These quantum signatures and verifications constitute a chain structure in a transaction business. At the beginning of the multi-party transaction, trader A should send a request for the transaction, and no verification is requested. At the end of the multi-signature, block creator should verify the quantum signatures of all traders, and no quantum signature is required. Many multi-party transactions form a series of timestamp blocks connected by cryptographic hashes and distributed ledgers whose data are shared by all blocks. A successful transaction will be published on the whole blockchain for distributed ledgers, and each transaction ID can be traced back to every trader and quantum signature. The multi-party transaction model in Figure 1 is not only a chain of blocks, but also a chain of quantum signatures, so the multi-signature algorithm will easily impact the performance and scalability of blockchain.

As shown in Figure 1, the complexity of quantum multi-signature is more complicated than the single signature, where each trader in Figure 1 needs to verify the previous quantum signature. Each trader is composed of transaction inputs and transaction outputs, where the inputs have to be unspent outputs before quantum signing, and the outputs of previous transactions are not spent before verification. After verifying the hash value of a transaction ID and the quantum key of the previous trader, the current trader will sign the transaction and transfer the signed transaction message to the next trader or block creator. The public key cryptography is frequently used in the classic blockchain to provide a secure multi-party transaction, which is often based on all kinds of hard mathematical problems, such as the integer factorization and discrete logarithms. However, quantum computers are hoped to have a dramatic computing capability to solve these problems more efficiently than the classical ones [3,4]. In our framework, the cryptography and hash functions in the industrial blockchain are redesigned in Figure 1 to resist quantum attacks.

On the one hand, a complex multi-signature will greatly diminish the efficiency and salability of multi-party blockchain transactions. In Figure 1, the lattice-based signature scheme has advantages over the generic methods in efficiency and the size of parameters, and the Bonsai Trees technology can be used to expand a lattice space into multiple lattice spaces for the corresponding transaction keys [9,10,11,12]. Among them, each signature employs a lattice space to achieve the randomness and the security of keys supporting the blockchain applications in the post-quantum era. Complex blockchain transactions often require more than two parties in their trading process, which may result in a sharp deterioration of computing performance [16,17]. If a lattice-based multi-signature is directly applied in blockchain to provide anti-quantum signature schemes, there may be wallet bloat [28,29]. Hence, lattice-based multi-signature schemes that are promising to resist quantum attacks suffer from the size of the large public keys and the signatures, so they are unsuitable for multi-party blockchains.

On the other hand, the multi-party transaction is more vulnerable to quantum attacks than the single-party transaction. All blockchain traders in Figure 1 will work automatically without any arbitrator. However, most existing multi-signature schemes are based on the discrete logarithm problem, which is verified to be vulnerable to quantum attacks [3,29]. The reason why the blockchain technology is increasingly popular in recent times is its decentralized and distributed architecture to provide strong security and privacy protection. In a lattice-based multi-signature, any forger attacking blockchain transactions or multiple traders should solve the complex lattice problem, where the security is provided by the rejection sampling theory [16,17]. With the increasing number of blockchain traders, the anti-quantum security of lattice-based multi-signature algorithms may be decreased.

In Figure 1, the multi-signature scheme should secure multi-party blockchain transactions and does not impair the blockchain performance. Although quantum signature is a more promising method, any quantum signature methods with an arbitrator are not suitable for multi-party blockchain transactions [32]. Different from the classical anti-quantum blockchain frameworks, the proposed anti-quantum blockchain architecture in Figure 1 employs quantum multi-signature without any arbitrator. More importantly, the algorithm complexity is especially considered to keep the computational performance and scalability of blockchains.

### 3.2. Quantum Key Distribution in the Industrial Blockchain

To apply the quantum blind multi-signature method into blockchain multi-party transactions, some necessary adjustments are needed to provide efficient quantum key distribution. On the one hand, enough quantum keys should be generated to deliver transaction messages directly to traders or users to provide more secure service. On the other hand, the number of quantum keys should not be greatly increasing with the increasing number of blocks to significantly diminish the scalability of the whole blockchain.

Therefore, the proposed scheme in Figure 1 generates n quantum keys for n trading parties to provide a balance between the quantum security and blockchain scalability. Taking two traders for example, trader A, trader B, and block creator C are supposed to share a series of triplet particles belonging to A, B, and C, respectively, where each triplet state can be represented as $|\phi \rangle_{ABC}=\frac{1}{\sqrt{2}}(|000\rangle +|111\rangle {)}_{ABC}$. Assume that the blind transaction message is $R_{M}=\{R_{i}\}$, where $R_{i}=1$ or $R_{i}=0$ is represented as m=1 or m=−1, respectively. The quantum state that the trader needs to send is $|\phi \rangle_{R}=\frac{1}{\sqrt{2}}(|0\rangle +m|1\rangle {)}_{R}$.

At the start of the blockchain transaction, trader A performs a Bell state measurement on the qubit $R_{A}$ and announces its measurement results. Trader B and block creator C perform a unitary transformation on the particles B and C, respectively, according to the corresponding measurement results of trader A. For each transaction, the quantum states of all qubits form a quantum system, which is

(1) $$|\phi \rangle_{R}⊗|\phi \rangle_{ABC}=\frac{1}{\sqrt{2}}(|0\rangle +m|1\rangle {)}_{R}⊗\frac{1}{\sqrt{2}}(|000\rangle +|111\rangle {)}_{ABC}\;=\frac{1}{2}{[|\phi^{+}\rangle }_{RA}⊗\frac{1}{\sqrt{2}}(|00\rangle +m|11\rangle {)}_{BC}+|\phi^{-}\rangle_{RA}⊗\frac{1}{\sqrt{2}}(|00\rangle -m|11\rangle {)}_{BC}\;+|\phi^{\to }\rangle_{RA}⊗\frac{1}{\sqrt{2}}(m|00\rangle +|11\rangle {)}_{BC}+|\phi^{↑}\rangle_{RA}⊗\frac{1}{\sqrt{2}}(-m|00\rangle +|11\rangle {)}_{BC}$$

where $|\phi^{\pm }\rangle_{MA}=\frac{1}{\sqrt{2}}(|00\rangle \pm |11\rangle {)}_{RA}$, $|\phi^{\to ↑}\rangle_{MA}=\frac{1}{\sqrt{2}}(|01\rangle \pm |10\rangle {)}_{RA}$.

These entangled particles can be used to generate secure quantum keys for multiple traders to make multi-signatures and implement complex transactions, and the measurement of entangled quantum states of the traders can be guaranteed by quantum non-cloning theorem and Bell inequality. The qubit BC is in the following public state $|\phi \rangle_{BC}=\frac{1}{\sqrt{2}}(|00\rangle +m|11\rangle {)}_{BC}$. With the help of block creator C, once trader B knows the measurement result of block creator C, the initial state of the transaction message $R_{M}=\{R_{i}\}$ can be restored according to the corresponding transformation in Table 1. The transaction message $R_{M}=\{R_{i}\}$ will be transmitted to trader B with the help of block creator C by the proposed quantum key distribution in Table 1, and trader B can restore the transaction message by performing a transformation on the particles in his hand. For example, if the measurement result of block creator C is $|\sigma_{1}\rangle_{3}$, then the transformation of trader B is $I_{2}$; if the measurement result of block creator C is $|\sigma_{2}\rangle_{3}$, then the transformation of trader B is ${\left(\sigma_{3}\right)}_{2}$.

Based on Table 1, the blockchain framework in Figure 1 can provide efficient quantum multi-signatures to meet the requirements of multi-party transactions without an arbitrator. The quantum key distribution in Table 1 can help us build an efficient quantum multi-signature for multi-party transactions with the same number of quantum keys to traders, but the computational resources of classic algorithms will be a polynomial rise with the number of traders [28,29,32]. Supposing block creator C uses a new measurement base {|→〉,|↑〉}, where $\left|\to \rangle =\frac{1}{\sqrt{2}}(|0\rangle +|1\rangle \right)$, $\left|↑\rangle =\frac{1}{\sqrt{2}}(|0\rangle -|1\rangle \right)$, the common state of the qubit BC can be expressed as

(2) $$\left|\phi \rangle_{BC}=\frac{1}{\sqrt{2}}[\frac{1}{\sqrt{2}}(|0\rangle +m|1\rangle {)}_{B}|\to \rangle +\frac{1}{\sqrt{2}}(|0\rangle -m|1\rangle {)}_{B}|↑\rangle_{C}\right]$$

Assuming there are n qubits as a quantum key for the proposed anti-quantum blockchain, the space performance of the proposed method is O(n), and the computing performance is O(n). Therefore, the multi-signature architecture will be lightweight for secure multi-party blockchain transactions, and the scalability performance of industrial blockchain is a linear function of the length n of the quantum keys.

## 4. Algorithm Design

The quantum blind multi-signature method allows multiple traders to complete a multi-party transaction, but the message and the final signature are unknown to the traders. A series of quantum keys is generated and verified for block creation to provide quantum resistance [28,29]. The whole algorithm flow includes four phases, i.e., initialization, signing, verification, and implementation.

### 4.1. Initialization Phase

**Figure 2:**
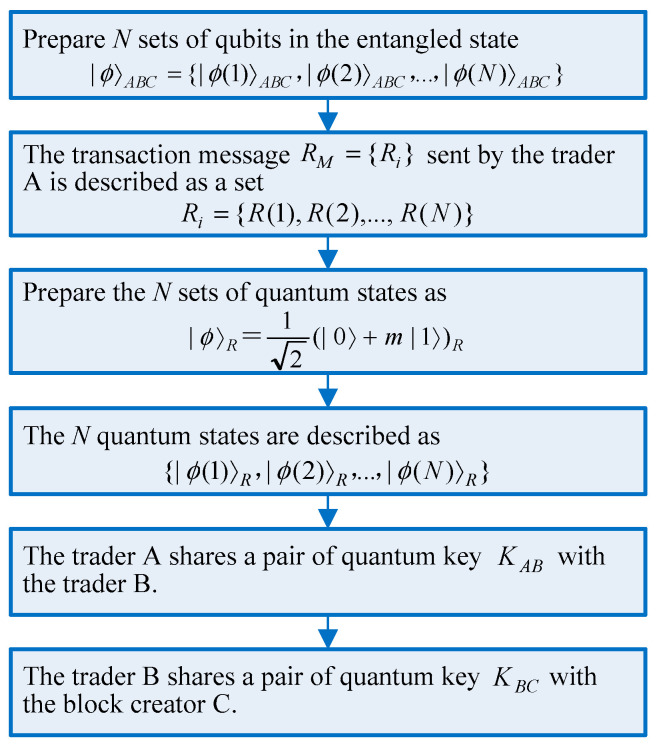
The initialization phase.

In this stage, the proposed algorithm prepares a series of quantum keys for quantum blind multi-signature scheme and multi-party blockchain transactions, in which block creator agrees on a common piece of information with the traders and blindly signed transaction messages. For a transaction message $R_{M}=\{R_{i}\}$ between trader A and trader B, the signatures of trader A and trader B on the message are denoted as $S_{A}$ and $S_{B}$, respectively. The transaction message $R_{M}=\{R_{i}\}$ sent by trader A is described as a set $R_{i}=\{R(1),R(2),\ldots ,R(N)\}$. Quantum key distribution can be performed on the parties A, B, and block creator C using Bell protocol. The initialization algorithm flow is shown in Figure 2.

Different from traditional blockchain business, the quantum key distribution should be built by negotiation in the initialization phase. Negotiation allows multiple traders to prepare secure quantum keys against malicious attack activities. The trade requesting party A at first prepares N sets of qubits in the entangled state $\left|\phi \rangle_{ABC}={\{|\phi (1)\rangle }_{ABC},|\phi (2)\rangle_{ABC},\ldots ,|\phi (N)\rangle_{ABC}\right\}$ for several traders to perform the quantum multi-signature. It is assumed that after negotiation, trader A shares a pair of quantum keys $K_{AB}$ with trader B, and trader B shares a pair of quantum keys $K_{BC}$ with block creator C. The transaction lets a trader A buy a product or service from a trader B or more traders. Trader A prepares the N sets of quantum states as $|\phi \rangle_{R}=\frac{1}{\sqrt{2}}(|0\rangle +m|1\rangle {)}_{R}$. They can help multiple traders make a contract between themselves where the sellers will not get paid until the buyer A obtains a product or service. Then, the N quantum states can be described as ${\{|\phi (1)\rangle }_{R},|\phi (2)\rangle_{R},\ldots ,|\phi (N)\rangle_{R}\}$. Multiple traders and block creator will determine whether the transaction terms are reasonable and feasible, and the negotiation of quantum keys will determine whether the quantum communication is being eavesdropped or the multi-party transaction is safe.

This phase will also provide secure negotiation for transaction terms and quantum keys. In any partially blind signature scheme, the trader is permitted to explicitly insert the common information in the signature based on negotiation, without violating the blindness property.

### 4.2. Signing Phase

**Figure 3:**
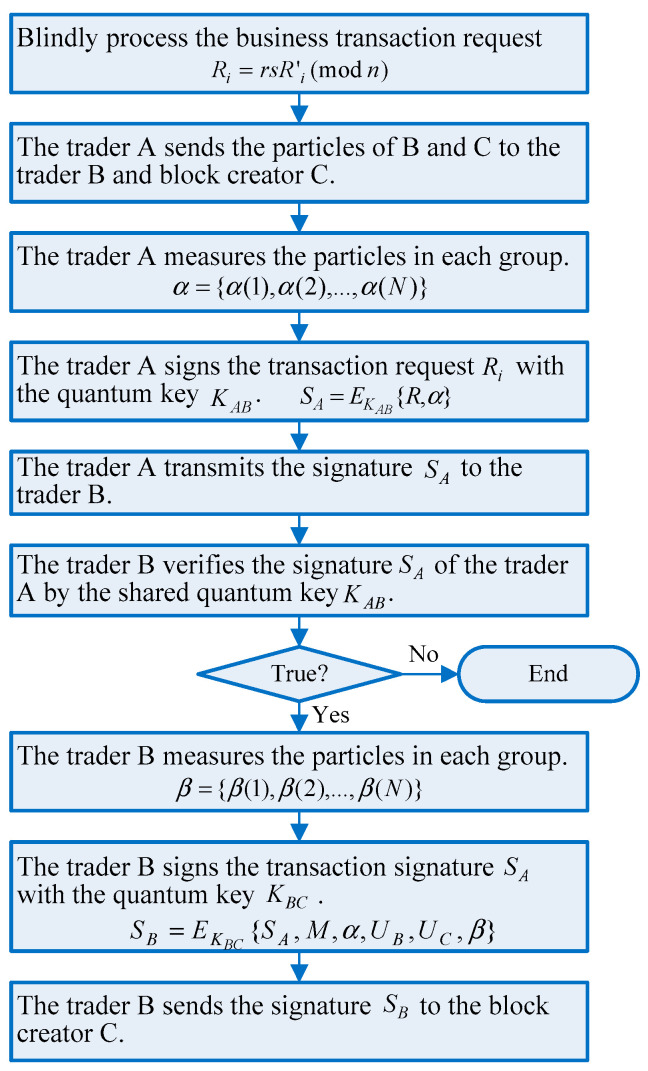
The signing phase.

Now, the transaction message should be blind before transactions, and each trader cannot know the specific content of the signature message during the signing process. The blinding factor r and the transaction summary information s are randomly selected to blindly process the business transaction request ${R^{\prime }}_{i}$. That is $R_{i}=rs{R^{\prime }}_{i}(\mathrm{mod}n)$. After quantum processing of the blind transaction message $R_{i}$ according to the Formula (1), trader A leaves his particles of each entangled particle group in his own hand, and sends the particles of B and C to trader B and block creator C. The signing algorithm flow is shown in Figure 3.

In the proposed framework, the system protects multiple traders against quantum attacks by quantum multi-signature method on a blockchain transaction before it can be considered as valid. This is realized by a series of quantum signatures where the minimum of n quantum keys is needed to sign a transaction before the tokens are spent. Trader A measures the particles in each group using a Bell basis and records the measurement results α={α(1),α(2),…,α(N)}, where $\alpha (i)\in \{|\phi^{+}\rangle ,|\phi^{-}\rangle ,|\phi^{\to }\rangle ,|\phi^{↑}\rangle \}$. If the comparison with the measurement results is invalid, the blockchain transaction will not be implemented and trader A cannot get a product or service and finish his payment.

Thereafter, if the measurement results are considered to be coincident, secure quantum keys $K_{AB}$ will be generated and trader A will encrypt the transaction request $R_{i}$ with the quantum key $K_{AB}$ and obtain the signature $S_{A}=E_{K_{AB}}\{R,\alpha \}$ of trader A, before trader A transmits the $S_{A}$ to trader B. At this point, trader A has completed the blind signature on the message $R_{i}$ according to the multi-party transaction, and trader B has also received the transaction request and the signature $S_{A}$ of trader A. However, trader B does not know the content of the blind signature message.

For the next trader B, after receiving the signature $S_{A}$ sent by trader A, trader B begins to perform a unitary transformation $U_{B}=\{U_{B}(1),U_{B}(2),\ldots ,U_{B}(N)\}$ on the particles of B in each set of entangled particles, and at the same time performs the other unitary transformation $U_{C}=\{U_{C}(1),U_{C}(2),\ldots ,U_{C}(N)\}$ on the particles of C according to the correspondence relationship in Table 1. In quantum multi-signature, the traders can separate discrete binary numbers into several fixed-length sets of bits. Trader B can verify the signature $S_{A}$ of trader A by the shared quantum key $K_{AB}$ with trader A. If the measurement error is greater than a predefined threshold, the signature is invalid, and the transaction will be discarded. If the measurement error fits the predefined requirement, then the signature will be taken as valid.

Then trader B measures each group of particles C with the specified measurement basis whose measurement result is $\beta =\{\beta (1),\beta (2),\ldots ,\beta (N)\}(\beta (i)\in |x_{1}\rangle ,|x_{2}\rangle \})$, and encrypts the transaction message with the key $K_{BC}$ before the signature $S_{B}=E_{K_{BC}}\{S_{A},M,\alpha ,U_{B},U_{C},\beta \}$ is obtained. To prevent the banks or traders or attackers from tracking the transaction message, all traders do not want the others to know the contents of their blind message (i.e., trader ID, the timestamp, and hash value), which is protected by blind signature technology. After the blind multi-signature is completed, trader B will send the quantum signature $S_{B}$ to block creator C to perform verification operation.

The cases with more traders can be analogized. Multiple traders can sign the blind message in turn and encode the signed transaction message in a prescribed format before sending it to blockchain for consensus testing over the classic channel.

### 4.3. Verification Phase

**Figure 4:**
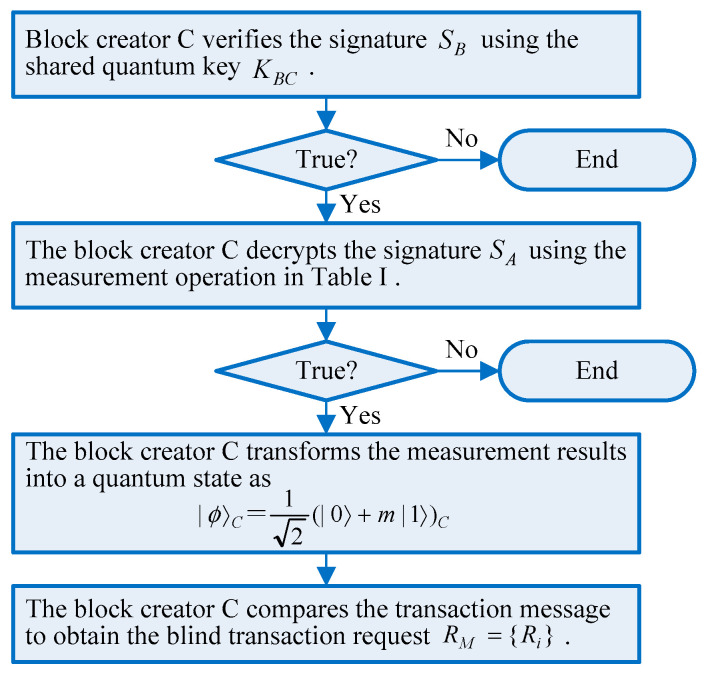
The verification phase.

In this phase, the coded transaction message is tested using a consensus mechanism and the signatures are verified, where all blocks will test the message successfully before reaching a consensus on the newly released transaction. Considering that trader A and trader B sign the same transaction message $R_{M}=\{R_{i}\}$, block creator C verifies the signatures of the traders A and B. The verification algorithm flow is shown in Figure 4.

If the trigger condition preset in the blockchain is met, the signed message will be tested and further determined whether it will be executed. After block creator C receives the signature $S_{B}$ and the particles sent by trader B, it will directly verify the authenticity of the signatures of trader B. The contract that is agreed upon after testing will be spread to different nodes in the whole network in a block manner. Then, block creator C will decrypt the signature $S_{B}$ using the shared quantum key $K_{BC}$, to obtain the blind transaction message and $\alpha (i),U_{B}(i),U_{C}(i)$ in each group, and judge whether the correspondence in Table 1 can be satisfied. If satisfied, block creator C will accept the signature $S_{B}$ of trader B. Otherwise, it will consider that the signature is forged and terminate the blockchain transaction.

After the signature verification of trader B is successful, block creator C will decrypt the signature $S_{A}$ using the measurement in Table 1 to obtain the blind transaction request $R_{M}=\{R_{i}\}$. Although everyone receives the blind message, the transaction information stays anonymous, and the messages sent to the recipients are decrypted by their quantum keys. According to the value of β(i) and the correspondence relationship of Table 1, block creator C performs a unitary transformation on the B particles in each group, and checks whether the qubit BC is correctly transformed into the public state shown in Formula (2) during the transaction. If the correspondence in Table 1 is satisfied by $\alpha (i),U_{B}(i),U_{C}(i)$ in each group, block creator C can judge that trader A has signed the transaction request $R_{i}$. Then block creator C will transform the measurement results into a quantum state as $|\phi \rangle_{C}=\frac{1}{\sqrt{2}}(|0\rangle +m|1\rangle {)}_{C}$.

Then, trader B uses the specified measurement basis to measure the particles of B in each group to obtain the value of m, and further derives the value of the blind transaction message $R_{i}$. Block creator C can compare the transaction message $R_{i}$. If the two are equal, the $S_{A}$ and $S_{B}$ will be accepted as the trusted signatures of traders A and B for the blind transaction message $R_{M}=\{R_{i}\}$. Otherwise, the blockchain transaction service will be abandoned. To satisfy the scalability and the memory capability of the multi-party blockchain, the messages are only stored for a short period of time before the consensus mechanism is finished.

The case with more traders can also be analogized. Then, the blockchain system will automatically complete the remaining transaction processes according to the predetermined procedure.

### 4.4. Implementation Phase

After the signature verification meets the requirements of the blockchain transaction, the blind message will be recovered to be a plaintext for implementation. That is, ${R^{\prime }}_{i}=r^{-1}R_{i}(\mathrm{mod}n)$. In the end phase, a blockchain transaction will be executed according to the transaction message ${R^{\prime }}_{i}$, which is de-blind to obtain the original transaction message. Every one of the traders can complete the multi-signature transaction according to different business requests, and each trader can add his signature to create a quantum signature script. After the verification, the blockchain will execute and complete the multi-party transaction in accordance with the business terms, and the successful transaction will be broadcasted to the whole blockchain as distributed ledgers.

At this point, block creator will publish the transaction message in the whole blockchain for consensus acknowledgment. If the transaction message is verified by the whole blockchain, it will be kept into accounts. Then, the hyperledger block will be created successfully by the hash value of the transaction. Once a trader signs a transaction message and broadcasts it to the whole blockchain network, all nodes will ensure that the signature script matches the redeem script hash provided by the blockchain. At last, the created hyperledger block will be added at the end of the existing blockchain, and the common accounting will be completed by all the distributed ledgers on each block, meaning the multi-party business is completed. Otherwise, the transaction will be automatically rejected.

### 4.5. Algorithm Summary

**Figure 5:**
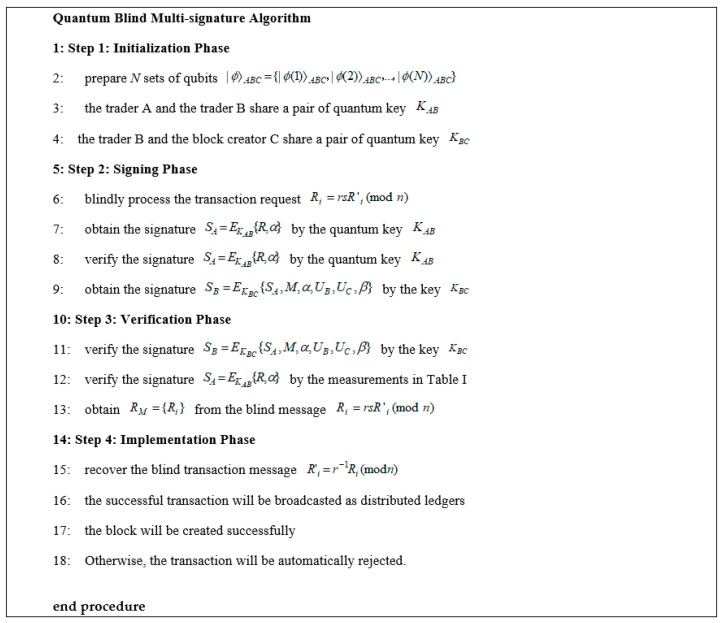
The proposed algorithm.

The whole algorithm flow to finish a quantum blind multi-signature transaction is summarized in Figure 5, where there are four main steps, including initialization, signing phase, verification, and implementation. Trader A prepares N sets of qubits in the entangled state $\left|\phi \rangle_{ABC}={\{|\phi (1)\rangle }_{ABC},|\phi (2)\rangle_{ABC},\ldots ,|\phi (N)\rangle_{ABC}\right\}$ for multiple traders to perform quantum multi-signature and block creator to complete verification. The transaction message $R_{M}=\{R_{i}\}$ sent by trader A is described as a set $R_{i}=\{R(1),R(2),\ldots ,R(N)\}$. Then the blinding factor r and the transaction summary s are randomly selected to blindly process the transaction message ${R^{\prime }}_{i}$. That is, $R_{i}=rs{R^{\prime }}_{i}(\mathrm{mod}n)$. After that, trader A and trader B (or more traders) sign the same transaction message $R_{M}=\{R_{i}\}$, and block creator begins to verify their signatures. The algorithm flow employs quantum key distribution instead of a complex algorithm to provide quantum resistance for multi-party transactions in the blockchain.

As we can see from Figure 5, the proposed quantum blind multi-signature method is different from traditional lattice-based anti-quantum signature algorithms [9,10,11,12] or other quantum single-signature algorithms [13,14,15]. First, it comprises the quantum signature, blind signature, and multi-signature into blockchain transactions to provide secure multi-party transaction for more than two traders, as shown in Figure 1. Second, the quantum key distribution is lightweight, where the trader obtains the signature $S_{A}=E_{K_{AB}}\{R,\alpha \}$ by the quantum key $K_{AC}$, and trader B obtains the signature $S_{B}=E_{K_{BC}}\{S_{A},M,\alpha ,U_{B},U_{C},\beta \}$ by the key $K_{BC}$. The number of quantum keys is in accordance with the number of traders, so the signing phase and the verification phase are easy to implement with good computational performance. Third, the proposed method can provide good scalability for the multi-party blockchain transaction. That is, the number of quantum keys and the signature computation in a blockchain transaction is a linear function of the number of the traders. Fourth, comparing with classic anti-quantum signature methods, the proposed algorithm can provide better computational performance for a light-weighted block node. To sum up, for x traders, the space performance of the proposed algorithm for quantum keys is O(x), and the computational performance for signing and verification is also O(x).

## 5. Performance Analysis

### 5.1. Security Analysis

Different from typical multi-signature method with a trusted arbitration mechanism, the validity of the proposed multi-signature is checked by the quantum non-cloning theorem, and the legitimacy of the multi-party transaction is mutually checked by all nodes without any arbitrator. The signers or attackers cannot forge any legal signatures in the proposed blockchain framework.

**Lemma** **1.** *Any trader cannot get the other traders’ quantum signatures.*

**Proof** **of** **Lemma** **1.** It is assumed that trader D wants to achieve the key $K_{AB}$ shared by trader A and trader B through a quantum entangle attack. That is, trader D performs a unitary measurement operation $\hat{U}$ on every particle of $S_{A}$ with an auxiliary quantum system |ϕ〉. Without loss of generality, the operation $\hat{U}$ can be expressed as

(3) $$\hat{U}(|0\rangle |\phi \rangle )=\alpha |0\rangle |\phi^{+}\rangle +\beta |1\rangle |\phi^{-}\rangle$$

(4) $$\hat{U}(|1\rangle |\phi \rangle )=\alpha^{\prime }|0\rangle |\phi^{\to }\rangle +\beta^{\prime }|1\rangle |\phi^{↑}\rangle$$

Here, $\hat{U}$ is a unitary operation in Hilbert space and abides by the rule ${\hat{U}}^{+}\hat{U}=I$. According to the quantum non-cloning theorem, any attacker cannot obtain legal quantum keys by cloning, entanglement, copying, measuring, etc. Since the particles of A, B, and D are in their own hands, the measured results of the attacker, namely trader D, can be given as

(5) $$|\alpha {|}^{2}\langle \phi^{+}|\phi^{+}\rangle +|\beta {|}^{2}\langle \phi^{-}|\phi^{-}\rangle =1$$

(6) $$|\alpha^{\prime }{|}^{2}\langle \phi^{\to }|\phi^{\to }\rangle +|\beta^{\prime }{|}^{2}\langle \phi^{↑}|\phi^{↑}\rangle =1$$

According to quantum mechanics, a particle |0〉 or |1〉 in signature $S_{A}$ can keep unchanged after the measurement operation $\hat{U}$ of a legal receiver. On the contrary, after the measurement of the trader D, this particle shared by trader A and trader B has a certain possibility of being state collapse, which will result in larger measurement error to be easily detected by trader A and block creator C. The unitary operation can be described as

(7) $$\hat{U}(|0\rangle |\phi \rangle )=|0\rangle |\phi^{+}\rangle$$

(8) $$\hat{U}(|0\rangle |\phi \rangle )=|1\rangle |\phi^{↑}\rangle$$

That is to say, $\alpha =\beta^{\prime }=1$, $\alpha^{\prime }=\beta =0$. It is impossible. Therefore, any trader cannot obtain the other’s quantum signature. □

**Lemma** **2.** *Any attackers cannot forge a transaction message by intercept-resend quantum attacks.*

**Proof** **of** **Lemma** **2.** By Lemma 1, any attacker cannot get the legal quantum keys.

The blind transaction message $R_{M}=\{R_{i}\}$ and the signature $S_{A}$ of trader A are encrypted by $K_{AB}$ in the transaction, and the blind transaction message $R_{M}=\{R_{i}\}$ and $S_{B}$ of trader B are encrypted by $K_{BC}$.

Since the particles of A, B, and C are in their own hands, according to the quantum non-cloning theorem, the attackers cannot forge any blind message or multi-signatures by intercept-resend quantum attacks due to the unconditional security of the entangled keys $K_{AB}$ and $K_{BC}$. □

**Lemma** **3.** *The attacker and other traders cannot forge a transaction by man-in-the-middle (MITM) quantum attacks.*

**Proof** **of** **Lemma** **3.** According to Lemmas 1 and 2, it is impossible for an attacker to counterfeit a legal trader (i.e., trader B). Hence, trader B (i.e., the attacker) cannot forge the encrypted transaction message $R_{i}$ and β.

The forged {UB, UC} by trader B (i.e., the attacker) will not conform to the entanglement characteristic of the quantum keys shared by trader B and block creator C. Since the particles of A, B, and C are in their own hands, the attacker cannot forge the signature $S_{B}$ of trader B and the signature $S_{A}$ of trader A.

Due to the quantum non-cloning theorem, the attacker cannot counterfeit trader B to obtain the $K_{AB}$ to falsify a transaction message by operations such as cloning, entanglement, copying, and measurement.

It is assumed the attacker falsifies the man-in-the-middle attacker (i.e., trader B) to sign the transaction message. According to the proposed quantum blockchain, the fake signature will be performed by the multi-signature transformation in Table 1, so the Equations (7) and (8) can be further transformed as

(9) $$\hat{U}(|+\rangle |\phi \rangle )=\hat{U}[\frac{1}{\sqrt{2}}(|0\rangle +|1\rangle )|\phi \rangle ]=\frac{1}{\sqrt{2}}[\hat{U}(|0\rangle |\phi \rangle )+\hat{U}(|1\rangle |\phi \rangle )]=\frac{1}{\sqrt{2}}(|0\rangle |\phi^{+}\rangle -|1\rangle |\phi^{↑}\rangle )\;=\frac{1}{2}[|+\rangle (|\phi^{+}\rangle +|\phi^{↑}\rangle )+|-\rangle (|\phi^{+}\rangle -|\phi^{↑}\rangle )]$$

(10) $$\hat{U}(|-\rangle |\phi \rangle )=\hat{U}[\frac{1}{\sqrt{2}}(|0\rangle -|1\rangle )|\phi \rangle ]=\frac{1}{\sqrt{2}}[\hat{U}(|0\rangle |\phi \rangle )-\hat{U}(|1\rangle |\phi \rangle )]=\frac{1}{\sqrt{2}}(|0\rangle |\phi^{+}\rangle -|1\rangle |\phi^{↑}\rangle )\;=\frac{1}{2}[|+\rangle (|\phi^{+}\rangle -|\phi^{↑}\rangle )+|-\rangle (|\phi^{+}\rangle +|\phi^{↑}\rangle )]$$

In a legal blockchain transaction, a particle |+〉, |−〉 in $S_{A}$ will not introduce a greater error when it is measured by block creator C, it will keep the states |+〉 and |−〉. After the illegal measurement of the attacker on $S_{A}$, there will be a higher possibility to be discovered if the quantum state of this particle changes. Therefore, block creator C will get a wrong measurement result with high probability, that is

(11) $$\hat{U}(|+\rangle |\phi \rangle )=\hat{U}[\frac{1}{\sqrt{2}}(|0\rangle +|1\rangle )|\phi \rangle ]=\frac{1}{\sqrt{2}}[\hat{U}(|0\rangle |\phi \rangle )+\hat{U}(|1\rangle |\phi \rangle )]=\frac{1}{\sqrt{2}}(|0\rangle |\phi^{+}\rangle +|1\rangle |\phi^{↑}\rangle )=\frac{1}{2}|+\rangle (|\phi^{+}\rangle +|\phi^{↑}\rangle )$$

(12) $$\hat{U}(|-\rangle |\phi \rangle )=\hat{U}[\frac{1}{\sqrt{2}}(|0\rangle -|1\rangle )|\phi \rangle ]=\frac{1}{\sqrt{2}}[\hat{U}(|0\rangle |\phi \rangle )-\hat{U}(|1\rangle |\phi \rangle )]=\frac{1}{\sqrt{2}}(|0\rangle |\phi^{+}\rangle -|1\rangle |\phi^{↑}\rangle )\;=\frac{1}{2}|-\rangle (|\phi^{+}\rangle +|\phi^{↑}\rangle )$$

From Equations (11) and (12), it can be known that an auxiliary system |ϕ〉 will be in a new state $\frac{1}{2}(|\phi^{+}\rangle +|\phi^{↑}\rangle )$ after an illegal measurement operation $\hat{U}$ is performed by |+〉 or |−〉. Thus, the attacker cannot determine whether an auxiliary system |ϕ〉 successfully performs a legal signature with a corresponding state by attacking measurement operation $\hat{U}$. Then, the attacker cannot get any useful information about the legal signature $S_{A}$ of trader A by the measurement operation $\hat{U}$ without being detected.

Hence, this falsified signature will be detected by block creator C and the transaction cannot be performed successfully. That is, the man-in-the-middle quantum attack will fail. □

**Lemma** **4.** *Multiple signers cannot deny their signatures.*

**Proof** **of** **Lemma** **4.** Taking two traders for example, the two signatures $S_{A}$ and $S_{B}$ of the blockchain transaction scheme use the key $K_{AB}$ shared by trader A and trader B, and the key $K_{BC}$ shared by trader B and block creator C, respectively, abides by the quantum mechanics.

By the non-cloning theorem of quantum keys, the successfully verified signatures will automatically trigger the predefined conditions and release the transaction to all blocks on the blockchain. Then the entire blockchain network cannot deny the transaction and their signatures.

Since the particles of A, B, and C are in their own hands, after the signature of the first trader A, the blind transaction message will be sent to trader B and block creator C in turn. After that, trader B makes his signature $S_{B}$ to perform a specified unitary transformation on the particle B, and then block creator C also performs a recovery operation on the signatures $S_{A}$ and $S_{B}$ to obtain the initial state of the transaction message $R_{i}$. Therefore, trader B cannot deny the non-recovery message after sending the blind message to block creator C, and block creator C will use the shared quantum keys to verify the signatures $S_{A}$ and $S_{B}$, and cannot deny the signatures of trader A and trader B.

Therefore, after the transaction publication and the hyperledger finishing, traders A and B and block creator C cannot deny the received signatures. □

### 5.2. Comparison and Discussion

**Figure 6:**
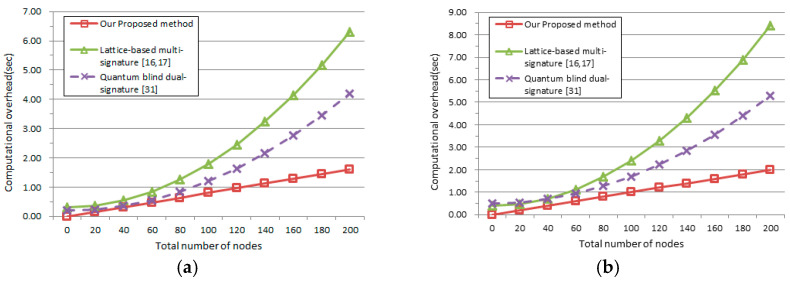
Computational overhead comparison. (**a**) Performance comparison of signing and (**b**) performance comparison of verification.

Here, the computational performance of the quantum key distribution and signature process are compared by computer simulation. The main compared indexes include computational overhead of the signature and verification, with the increasing number of blockchain nodes, as shown in Figure 6a,b. The lattice-based multi-signature [16,17] are quantum blind dual-signature [31] methods are selected as typical samples for comparison.

In the computer simulation of Figure 6, the quantum blind dual-signature [31] and our proposed quantum blind multi-signature scheme have advantages over the lattice-based multi-signature method [16,17] in both the signing stage and the verification stage. Our proposed method can keep the signing performance and verification performance when the total number of nodes is increasing from 0 to 200. However, the computational overhead of the other two methods will be a rising polynomial. Therefore, our proposed method can provide better blockchain scalability than the quantum blind dual-signature [31] and the lattice-based multi-signature methods [16,17].

Furthermore, more signature algorithms are compared here, and the performance indicators for comparison include the quantum intercept-resend (QIR) attacks, quantum man-in-the-middle (QMITM) attacks, blind message, number of signatures, signature complexity, and verification complexity. The compared schemes include the lattice-based signature [10,11,12], lattice-based blind signature [9,26], lattice-based multi-signature [16,17], quantum signature [13], quantum Fourier transfer [14], quantum blind signature [15], arbitrated quantum blind dual-signature [31], and our proposed framework in this paper. It is assumed that p is a prime in a k-dimensional lattice with m elements, where m=poly(k). Assuming there are n qubits to form a quantum key for quantum signature or n bits to form a classic key for classic signature, the comparison results of different signature algorithms are shown in Table 2.

Based on the above comparison results, we can see that:

(1) Facing the security threaten from quantum technologies [3,4], the proposed framework can provide absolute anti-quantum security through the quantum non-cloning theorem. However, the classic anti-quantum technologies [9,10,11,12,16,17,26] can only provide probabilistic quantum resistance with complex algorithms.
(2) Our proposed method, the lattice-based multi-signature scheme [16,17] and the arbitrated quantum blind dual-signature [31] model can provide multi-signature operation for multi-party transactions in a blockchain. Nevertheless, the other schemes can only provide a single signature [9,10,11,12,13,14,15,26] and the arbitrated quantum blind dual-signature [31] model is unsuitable for multi-party transactions in industrial blockchains.
(3) Our proposed scheme, the classic blind signature schemes [9,26], and quantum blind signature methods [15,31] use blind operation on the transaction message, and can be used for privacy protection of multi-party transactions in a blockchain. However, other methods [10,11,12,13,14,16,17] cannot provide blind privacy protection.
(4) Compared with the classic anti-quantum schemes [9,10,11,12,16,17,26] based on solving complexity and other quantum signature algorithms [13,14,15,31], our proposed method can provide both absolute anti-quantum security and good computational performance for multi-party transactions with more than two traders. When the number of traders is increasing, the computational performance and scalability of the proposed multi-signature method will not greatly deteriorate. The proposed lightweight architecture is suitable for the decentralization blockchain architecture and provides good scalability.

## 6. Conclusions and Future Work

Industrial blockchains are expected to use anti-quantum technology in the post-quantum era to establish secure transactions for users and to resist quantum attacks. Here, we introduced a blockchain framework based on the quantum blind multi-signature model without an arbitrator. The multi-signature algorithm for a multi-party transaction includes four main steps, i.e., initialization, signing, verification, and implementation. Trader A prepares N sets of qubits $\left|\phi \rangle_{ABC}={\{|\phi (1)\rangle }_{ABC},|\phi (2)\rangle_{ABC},\ldots ,|\phi (N)\rangle_{ABC}\right\}$ in the entangled state for multiple traders to perform quantum multi-signature and block creator to implement verification. The transaction message $R_{M}=\{R_{i}\}$ sent by trader A is blind, where the blinding factor r and the transaction summary s are randomly selected to blindly process the transaction message $R_{i}=rs{R^{\prime }}_{i}(\mathrm{mod}n)$. Multiple traders will verify the previous signatures and sign the same transaction message $R_{M}=\{R_{i}\}$ by their own until the block creator verifies their signatures. The algorithm flow employs quantum signatures to provide quantum resistance for multi-party transactions in an industrial blockchain. The underlying framework and transaction algorithm of blockchain are lightweight and have good computational performance. Performance analysis confirms that our approach can provide privacy protection, unconditional security, and good scalability for multi-party blockchain transactions, which cannot be offered by the other compared methods.

For future research directions, the impact of the measurement error on the validity of quantum multi-signatures will be investigated. Additionally, the effects of quantum denial of service attacks and other quantum attacks on blockchain transactions will also be analyzed in future studies.

## Figures and Tables

**Table 1:** The transformation table for quantum key distribution.

The Measurement Results of Trader A	Transformation of Trader B/Block Creator C
$|\phi^{+}\rangle_{RA}$	$I_{2}⊗I_{3}$
$|\phi^{-}\rangle_{RA}$	$I_{2}⊗(\sigma_{3})I_{3}$
$|\phi^{\to }\rangle_{RA}$	$(\sigma_{1})I_{2}⊗(\sigma_{3})I_{3}$
$|\phi^{↑}\rangle_{RA}$	${\left(\sigma_{1}\right)}_{2}⊗{\left(\sigma_{3}\sigma_{1}\right)}_{3}$

**Table 2:** The comparative analysis of different secure schemes.

Model	QIR Attacks	QMITM Attacks	Blind Message	Number of Signatures	Signature Complexity	Verification Complexity
Lattice-based signature [10,11,12]	Probabilistic	Probabilistic	No	1	O(mknlogp)	$O(m^{2}n\log p)$
Lattice-based blind signature [9,26]	Probabilistic	Probabilistic	Blind	1	O(mknlogp)	$O(m^{2}n\log p)$
Lattice-based multi-signature [16,17]	Probabilistic	Probabilistic	No	≥2	O(mknlogp)	$O(m^{2}n\log p)$
Quantum signature [13]	Non-cloning	Non-cloning	No	1	O(n)	O(n)
Quantum Fourier transfer [14]	Non-cloning	Non-cloning	Blind	1	$O(n^{2})$	$O(n^{2})$
Quantum blind signature [15]	Non-cloning	Non-cloning	Blind	1	$O(n^{2})$	$O(n^{2})$
Quantum blind dual-signature [31]	Non-cloning	Non-cloning	Blind	2	$O(n^{2})$	$O(n^{2})$
Our proposed method	Non-cloning	Non-cloning	Blind	≥2	O(n)	O(n)

## Data Availability

No new data were created or analyzed in this study. Data sharing is not applicable to this article.

## References

[1] Liang W., Fan Y., Li K.C., Zhang D., Gaudiot J.L. (2020). Secure data storage and recovery in industrial blockchain network en-vironments. IEEE Trans. Ind. Inform..

[2] Li Z., Kang J., Yu R., Ye D., Deng Q., Zhang Y. (2017). Consortium Blockchain for Secure Energy Trading in Industrial Internet of Things. IEEE Trans. Ind. Inf..

[3] Fedorov A.K., Kiktenko E., Lvovsky A.I. (2018). Quantum computers put blockchain security at risk. Nature.

[4] Arute F., Arya K., Babbush R., Bacon D., Bardin J.C., Barends R., Biswas R., Boixo S., Brandao F.G.S.L., Buell D.A. (2019). Quantum supremacy using a programmable superconducting processor. Nature.

[5] Fernandez-Carames T.M., Fraga-Lamas P. (2020). Towards Post-Quantum Blockchain: A Review on Blockchain Cryptography Resistant to Quantum Computing Attacks. IEEE Access.

[6] Sun X., Kulicki P., Sopek M. (2021). Logic Programming with Post-Quantum Cryptographic Primitives for Smart Contract on Quantum-Secured Blockchain. Entropy.

[7] Gao Y.-L., Chen X.-B., Chen Y.-L., Sun Y., Niu X.-X., Yang Y.-X. (2018). A Secure Cryptocurrency Scheme Based on Post-Quantum Blockchain. IEEE Access.

[8] Mesnager S., Sınak A., Yayla O. (2020). Threshold-Based Post-Quantum Secure Verifiable Multi-Secret Sharing for Distributed Storage Blockchain. Mathematics.

[9] Li C., Xu G., Chen Y., Ahmad H., Li J. (2019). A New Anti-Quantum Proxy Blind Signature for Blockchain-Enabled Internet of Things. Comput. Mater. Contin..

[10] Di J., Xie T., Fan S., Jia W., Fu S. An Anti-Quantum Signature Scheme Over Ideal Lattice in Blockchain. Proceedings of the 2020 International Symposium on Computer Engineering and Intelligent Communications (ISCEIC).

[11] Li C.-Y., Chen X.-B., Chen Y.-L., Hou Y.-Y., Li J. (2018). A New Lattice-Based Signature Scheme in Post-Quantum Blockchain Network. IEEE Access.

[12] Tianyuan X.I.E., Haoyu L.I., Yiming Z.H.U., Yanbin P.A.N., Zhen L.I.U., Zhaomin Y.A.N.G. (2020). FatSeal: An efficient lattice-based signature algorithm. J. Electron. Inf. Technol..

[13] Tianyuan X.I.E., Haoyu L.I., Yiming Z.H.U., Yanbin P.A.N., Zhen L.I.U., Zhaomin Y.A.N.G. (2019). A novel e-payment protocol implented by blockchain and quantum sig-nature. Int. J. Theor. Phys..

[14] Lou X., Tang W., Long H., Cheng Y. (2019). A Quantum Blind Signature Scheme Based on Block Encryption and Quantum Fourier Transfer. Int. J. Theor. Phys..

[15] Cai Z., Qu J., Liu P., Yu J. (2019). A Blockchain Smart Contract Based on Light- Weighted Quantum Blind Signature. IEEE Access.

[16] Aitzhan N.Z., Svetinovic D. (2016). Security and privacy in decentralized energy trading through multi-signatures, blockchain and anonymous messaging streams. IEEE Trans. Dependable Secur. Comput..

[17] Xiao Y., Zhang P., Liu Y. (2020). Secure and Efficient Multi-Signature Schemes for Fabric: An Enterprise Blockchain Platform. IEEE Trans. Inf. Forensics Secur..

[18] Li T., Wang H., He D., Yu J. (2020). Permissioned Blockchain-Based Anonymous and Traceable Aggregate Signature Scheme for Industrial Internet of Things. IEEE Internet Things J..

[19] Wang S., Ouyang L., Yuan Y., Ni X., Han X., Wang F.Y. (2019). Blockchain-enabled smart contracts: Architecture, applications, and future trends. IEEE Trans. Syst. Man Cybern. Syst..

[20] Sengupta J., Ruj S., Das Bit S. (2019). A Comprehensive Survey on Attacks, Security Issues and Blockchain Solutions for IoT and IIoT. J. Netw. Comput. Appl..

[21] Zhu S., Li W., Li H., Tian L., Luo G., Cai Z. (2018). Coin Hopping Attack in Blockchain-Based IoT. IEEE Internet Things J..

[22] Iftekhar A., Cui X., Tao Q., Zheng C. (2021). Hyperledger Fabric Access Control System for Internet of Things Layer in Blockchain-Based Applications. Entropy.

[23] Aggarwal D., Brennen G.K., Lee T., Santha M., Tomamichel M. (2018). Quantum attacks on Bitcoin, and how to protect against them. Ledger.

[24] Stewart I., Ilie D., Zamyatin A., Werner S., Torshizi M.F., Knottenbelt W.J. (2018). Committing to quantum resistance: A slow defence for Bitcoin against a fast quantum computing attack. R. Soc. Open Sci..

[25] Chehelcheshmeh S.B., Hosseinzadeh M. (2016). Quantum-resistance authentication in centralized cognitive radio networks. Secur. Commun. Netw..

[26] Zhu H., Tan Y.-A., Zhu L., Wang X., Zhang Q., Li Y. (2018). An Identity-Based Anti-Quantum Privacy-Preserving Blind Authentication in Wireless Sensor Networks. Sensors.

[27] Persichetti E., Steinwandt R., Suárez Corona A. (2019). From Key Encapsulation to Authenticated Group Key Establishment—A Compiler for Post-Quantum Primitives. Entropy.

[28] He H., Lara-Avila S., Kim K.H., Fletcher N., Rozhko S., Bergsten T., Eklund G., Cedergren K., Yakimova R., Park Y.W. (2019). Polymer-encapsulated molecular doped epigraphene for quantum resistance metrology. Metrologia.

[29] Suhail S., Hussain R., Khan A., Hong C.S. (2020). On the Role of Hash-Based Signatures in Quantum-Safe Internet of Things: Current Solutions and Future Directions. IEEE Internet Things J..

[30] Cai Z., Zhang Y., Wu M., Cai D. (2015). An Entropy-Robust Optimization of Mobile Commerce System Based on Multi-agent System. Arab. J. Sci. Eng..

[31] Sun H.-W., Zhang L., Zuo H.-J., Zhang K.-J., Ma C.-G. (2018). Offline Arbitrated Quantum Blind Dual-Signature Protocol with Better Performance in Resisting Existential Forgery Attack. Int. J. Theor. Phys..

[32] Ferrer-Gomila J.-L., Hinarejos M. (2021). A Multi-Party Contract Signing Solution Based on Blockchain. Electronics.

